# Overexpression of an Alfalfa Gene, *MsCKX5*, Confers Cold Tolerance in Transgenic *Arabidopsis thaliana*

**DOI:** 10.3390/genes17050557

**Published:** 2026-05-06

**Authors:** Lin Bian, Xiaowei Huo, Qi Chen, Yan Zhang, Na Guo, Jing Xu, Jianwei Li, Zhiqiang Zhang

**Affiliations:** Key Laboratory of Grassland Resources of the Ministry of Education, Engineering Center of Drought and Cold-Resistant Grass Breeding in the North of the National Forestry and Grassland Administration, Technology College of Grassland Science, Inner Mongolia Agricultural University, Hohhot 010010, China; 19847376825@163.com (L.B.);

**Keywords:** *Medicago sativa*, *MsCKX5*, cold tolerance, transcriptome, metabolome

## Abstract

Background: Cytokinin oxidase/dehydrogenase (CKX) can irreversibly degrade cytokinin, regulating the growth and development of plants and helping them cope with environmental stress. Methods: To understand the expression characteristics and the biological functions of *CKX* in alfalfa (*Medicago sativa*), a novel gene, designated as *MsCKX5* (GenBank: PV934228), was cloned, characterized and overexpressed in *Arabidopsis thaliana*. Results: qRT-PCR results showed that *MsCKX5* gene expression was clearly tissue-specific and had the highest expression level in the stems. In addition, the expression level of *MsCKX5* was significantly induced by cold treatments. Heterologous expression of the *MsCKX5* gene in *A. thaliana* could enhance cold tolerance by regulating the activities of antioxidant enzymes such as superoxide dismutase (SOD), peroxidase (POD), and catalase (CAT), and adjust the relative conductivity and malondialdehyde (MDA) content. The combined analysis of transcriptome and metabolome results indicated that flavone and flavonol biosynthesis as well as the plant hormone signal transduction pathways were the main enriched pathways between wild-type and *MsCKX5* overexpressed lines. Conclusions: These results provide an important molecular basis for further elucidating the molecular mechanism of plant cold resistance and the breeding of cold-resistant crop varieties.

## 1. Introduction

Alfalfa (*Medicago sativa* L.) is a perennial leguminous forage species valued for its high nutritional quality and palatability [1]. It is widely cultivated in the cold and arid regions of northern China, including the northeast, north, and northwest. However, its growth and productivity are frequently constrained by abiotic stresses, among which low temperature has become a key factor restricting the safe overwintering of alfalfa and maintaining stable high yields [2]. Low-temperature stress exerts multifaceted effects on alfalfa, including disruption of cell membrane integrity, inhibition of photosynthesis and respiration, perturbation of metabolic homeostasis, and activation of the antioxidant defense system. These physiological changes directly impair the cold tolerance and overwintering ability of alfalfa [3,4,5].

Plant hormones play an essential role in mediating plant responses to various biotic and abiotic stresses [6]. As a class of phytohormones critically involved in plant growth and development, cytokinins (CTK) primarily exist in three active forms in higher plants: zeatin (Z), dihydrozeatin (DZ), and isopentenyladenine (iP) [7]. CTKs are implicated in nearly all aspects of plant growth and development, modulating processes ranging from cell division to nutrient allocation [8]. Exogenous application of CTKs has been shown to promote bud germination in various plant species [9,10]. Functionally, CTKs regulate multiple developmental processes, including the initiation of bud outgrowth, promotion of stem elongation, release of apical dominance, facilitation of chloroplast biogenesis, inhibition of root development, delay of leaf senescence, and mediation of nutrient signaling [11,12,13].

Cytokinin oxidase/dehydrogenase (CKX) is a key enzyme responsible for the irreversible degradation of endogenous cytokinins (CTK), thereby regulating CTK homeostasis in plants. It specifically cleaves unsaturated isoprenoid side chains, yielding adenine or adenosine along with the corresponding aldehydes, which leads to a reduction in the levels of biologically active CTKs [14,15]. To date, members of the *CKX* gene family have been identified in various plant species, mainly focusing on *Arabidopsis thaliana* [16], rice (*Oryza sativa*) [17,18], and soybean (*Glycine max*) [19]. Specifically, 7 *AtCKX* genes have been identified in *A. thaliana*, 11 *OsCKX* genes in rice, and 18 *GmCKX* genes in soybean. As a model system, *Arabidopsis* has been instrumental in elucidating *CKX* gene functions. Werner et al. demonstrated that *CKX* genes modulate cytokinin levels, thereby influencing root apical meristem activity, lateral bud development, and leaf senescence [16]. Bartrina et al. further showed that *CKX3* and *CKX5* regulate reproductive meristem activity and seed yield through cytokinin modulation [20]. In rice, functional studies have revealed that mutants targeting various *OsCKX* genes exhibit marked phenotypic variation in key agronomic traits, including leaf area, stem diameter, plant height, tiller number, and grain weight [18]. As core metabolic enzymes maintaining CTK homeostasis, *CKX* genes also play a role in regulating plant branching. For instance, the tiller number was significantly reduced in the *CKX1* and *CKX2* double mutants, whereas the *CKX4* and *CKX9* double mutants exhibited a notable increase in tiller number, consistent with elevated endogenous CTK levels [18].

Beyond developmental regulation, *CKX* genes are involved in plant responses to environmental stress. Reducing endogenous CTK levels has been shown to enhance stress tolerance. In *Arabidopsis*, both knockout of *OsIPT*s and overexpression of *OsCKX*s led to reduced CTK accumulation and improved resistance to salt and drought stress [21]. Li et al. reported that heterologous overexpression of an alfalfa-derived *MsCKX* gene (MK177192) in *Arabidopsis* enhanced plant tolerance to salt stress [22]. Additionally, Macková et al. employed the WRKY6 promoter to drive root-specific expression of AtCKX1 in *Arabidopsis*. Under stress conditions, early downregulation of WRKY6:*CKX1* resulted in elevated CTK concentrations, thereby improving plant resistance to drought and high-temperature stress [23].

Our previous studies have shown that *MsCKX5* is significantly induced by low-temperature stress, suggesting its potential involvement in the cold resistance response of alfalfa [24]. Accordingly, the present study aims to characterize the function of *MsCKX5* through gene cloning, expression profiling, and functional validation under cold stress conditions. Furthermore, integrated transcriptomic and metabolomic analyses were conducted to compare wild-type and *MsCKX5*-overexpressing lines. This work seeks to elucidate the role of *MsCKX5* in the cold defense mechanisms of alfalfa and to provide a theoretical basis for understanding the molecular underpinnings of cold tolerance in this species.

## 2. Materials and Methods

### 2.1. Plant Materials and Treatments

Plump and uniform seeds of alfalfa (*M. sativa* L. cv. ‘Zhongmu No. 1’) were selected and surface-sterilized in a 2% (*v*/*v*) sodium hypochlorite solution for 10 min with continuous agitation. The seeds were subsequently rinsed 5–6 times with distilled water and soaked in distilled water for 12 h. Following disinfection, the seeds were sown in plastic pots filled with a mixture of nutrient soil and vermiculite (2:1, *v*/*v*) and placed in an artificial climate chamber under long-day conditions (16 h light/8 h dark) with regular watering. After 45 days of growth, the seedlings were subjected to low-temperature treatment at 4 °C, and leaf samples were collected at 0, 4, 8, 12, and 24 h post-treatment for RNA extraction. For tissue-specific expression analysis, root, stem, leaf, flower, and seed samples were harvested during the podding stage. All samples were collected from three biological replicates, immediately frozen in liquid nitrogen, and stored at −80 °C until further use.

Seeds of wild-type (CK) and *MsCKX5*-overexpressing *Arabidopsis* lines (OE-5-2 and OE-5-4) were placed in 1.5 mL sterile RNase-free centrifuge tubes and surface-sterilized in a 5% (*v*/*v*) sodium hypochlorite solution containing 0.1% (*v*/*v*) Triton X-100 for 10 min with gentle agitation. The seeds were then rinsed five to six times with sterile distilled water and sown on 1/2-strength Murashige and Skoog (1/2 MS) solid medium. After stratification at 4 °C for 2 d in the dark, the plates were transferred to a plant growth chamber set at 23 °C under long-day conditions (16 h light/8 h dark). Following 14 d of growth, the seedlings were transplanted into plastic pots filled with a mixture of vermiculite and pumice (2:1, *v*/*v*) and placed in an artificial climate chamber under the same long-day conditions with regular watering.

### 2.2. Test Method

#### 2.2.1. Cloning of Target Gene and Construction of Plant Overexpression Vectors

Total RNA was extracted from ‘Zhongmu No. 1’ alfalfa and reverse-transcribed into cDNA, which served as the template for gene amplification. Specific primers for *MsCKX5* (Table 1) were designed using Primer 3.0 software, and PCR amplification was performed. The PCR products were stored at 4 °C, subsequently separated by agarose gel electrophoresis, and purified using a gel extraction kit (Tiangen Biotech, Beijing, China). The purified fragment was ligated into the pEASY-Blunt vector and transformed into *Escherichia coli* Trans1-T1 competent cells via heat shock. Positive clones were selected by colony PCR and confirmed by sequencing (Tsingke Biotechnology Co., Ltd., Beijing, China).

To construct the *MsCKX5* overexpression vector, specific primers containing EcoRI and BamHI restriction sites were designed based on the pCAMBIA1300-2×35S-EGFP vector map (Table 1). The *MsCKX5* coding sequence was amplified and inserted into the EcoRI-BamHI-digested pCAMBIA1300-2×35S-EGFP vector via homologous recombination. The ligation mixture was gently pipetted to mix, briefly centrifuged, and incubated at 50 °C for 30 min. The reaction was then placed on ice for 5 min and transformed into *E. coli* DH5α competent cells. Positive transformants were identified by colony PCR using gene-specific primers MsCKX5-F combined with vector-specific primer pCAMBIA1300-2×35S-EGFP-R (Table 1). Recombinant plasmids were extracted from positive clones and verified by 1% agarose gel electrophoresis. The validated plasmid was subsequently introduced into *Agrobacterium tumefaciens* strain GV3101 via heat shock. Positive colonies were confirmed by PCR, and the verified bacterial cultures were mixed with sterile 50% glycerol and stored at −80 °C for subsequent experiments.

#### 2.2.2. Bioinformation Analysis

The obtained nucleotide and deduced amino acid sequences of MsCKX5 were analyzed using multiple bioinformatics tools. Homology searches and sequence alignment were performed with the BLAST program on the NCBI website (https://blast.ncbi.nlm.nih.gov) (accessed on 7 December 2024). Conserved domains were identified using the NCBI Conserved Domain Database (CDD) in conjunction with BLAST. The primary physicochemical properties of the MsCKX5 protein, including molecular weight, theoretical isoelectric point, and amino acid composition, were predicted using the ProtParam tool on the ExPASy server (https://web.expasy.org/protparam/) (accessed on 9 December 2024). The secondary structure was predicted via the SOPMA online tool (https://npsa-prabi.ibcp.fr/cgi-bin/npsa_automat.pl?page=npsa%20_sopma.html) (accessed on 9 December 2024). A three-dimensional homology model of the MsCKX5 protein was generated using the SWISS-MODEL workspace (https://swissmodel.expasy.org/) (accessed on 11 December 2024), based on the closest structural templates identified.

#### 2.2.3. RT-qPCR Analysis

RT-qPCR primers were designed using the online software Primer 3.0 (Table 1). A total of 4 μL of cDNA template from different tissue sites, 0.4 μL of each forward and reverse primer, 5 μL of ddH_2_O, 0.2 μL of ROX Reference Dye (50×), and 10 μL of SYBR qPCR Master Mix (Jiangsu Cowin Biotech Co., Ltd., Taizhou, China) were used. The *β-actin* gene of alfalfa was used as the internal reference gene. Each sample was repeated 3 times, and the relative expression level of the gene was calculated using the 2^−∆∆Ct^ method [25]. The concentration of extracted RNA was detected with a NanoDrop (Thermo Fisher Scientific, Waltham, MA, USA) spectrophotometer, and RNA integrity was validated through PCR amplification. RT-qPCR assays were carried out on an ABI 7500 Real-Time PCR System (Applied Biosystems, Thermo Fisher Scientific, Waltham, MA, USA).

#### 2.2.4. Agrobacterium-Mediated *A. thaliana* Genetic Transformation

The floral dip method was adopted for the genetic transformation of *A. thaliana*. The agrobacterium tumefaciens suspension harboring *MsCKX5* was expanded for culture, and the bacterial precipitate was resuspended in an infiltration buffer (5% sucrose + 0.02% Silwet L-77, pH adjusted to 5.7) to a final OD_600_ of 0.8, yielding the agrobacterium resuspension. *A. thaliana* plants at the full flowering stage were selected, and their inflorescences were gently immersed in the agrobacterium resuspension for 30–60 s before being taken out. The infiltrated plants were covered with light-shading bags, incubated for 24 h in a dark environment, and then cultured under normal conditions until seed maturation. Genomic DNA was extracted and subjected to PCR identification. Positive plants with clear and bright amplified bands were selected for T_0_ generation seed harvesting.

#### 2.2.5. Phenotypic Identification of MsCKX5-Overexpressing *A. thaliana*

##### Method for Determination of Seed Vigor

Plump and uniformly sized *A. thaliana* seeds were selected as experimental materials. Seeds were surface-sterilized following the method described in Section 2.1. The sterilized seeds were sown on ½ MS solid medium, and all Petri dishes were placed in a light incubator for germination assays. The incubator was set to constant temperatures of 4 °C and 23 °C, respectively, to assess seed germination under different temperature conditions. The number of germinated seeds was recorded daily for a continuous period of 7 days.

##### Leaf Area Measurement Method

Seeds of T3 generation *MsCKX5*-overexpressing *Arabidopsis* lines (OE-5-2 and OE-5-4) and wild-type (CK) plants were surface-sterilized and sown following the method described in Section 2.1. After 4–5 weeks of growth, select the leaves from the same part of different plants and measure the leaf area using a leaf area meter. Each treatment has three biological replicates, and each replicate is measured three times.

#### 2.2.6. Measurement of Physiological Indicators

To assess membrane permeability, relative electrolyte leakage was measured. Fresh, mature leaves (0.1 g) harvested from the same position of plants at each treatment time point were rinsed with deionized water to remove surface contaminants. The leaf samples were placed in 15 mL heat-resistant centrifuge tubes containing 10 mL of deionized water. The tubes were subjected to vacuum infiltration for 30 min using a vacuum pump to ensure complete submersion of the leaves. Subsequently, the tubes were incubated on an orbital shaker at 25 °C and 100 rpm for 1.5 h. After incubation, the initial electrical conductivity (S1) of the solution was measured using a conductivity meter. The tubes were then placed in a boiling water bath (100 °C) for 10 min, allowed to cool to room temperature, and the final electrical conductivity (S2) was measured. Relative electrolyte leakage was calculated as (S1/S2) × 100%. For physiological analyses, leaf samples were collected from wild-type (CK) and *MsCKX5*-overexpressing *Arabidopsis* plants subjected to low-temperature treatment (4 °C) for 0 h and 24 h. The activities of antioxidant enzymes, including peroxidase (POD), superoxide dismutase (SOD), and catalase (CAT), as well as the malondialdehyde (MDA) content, were determined using corresponding assay kits according to the manufacturer’s instructions (Beijing Solarbio Science & Technology Co., Ltd., Beijing, China). All measurements were performed with three biological replicates.

#### 2.2.7. Transcriptome Sequencing Analysis

Leaves of CK, OE-5-2 and OE-5-4 grown for 45 days were subjected to transcriptome sequencing. The experiment was repeated in triplicate. RNA was extracted from the leaves using the Ultrapure RNA Kit (CWBIO, Taizhou, China), and the concentration and purity of the extracted RNA were assessed using a Nanodrop2000 (Thermo Fisher Scientific, United States). RNA purification, reverse transcription, library creation, and sequencing were performed by Majorbio Bio-Pharm Biotechnology Co., Ltd. (Shanghai, China). During the library construction process, the single library construction requires a total RNA amount of 1 μg, a concentration of ≥30 ng/μL, RQN > 6.5, and an OD260/280 ratio between 1.8 and 2.2.

To obtain high-quality clean reads, the sequencing quality was evaluated by analyzing the error rate, Q20, Q30, and GC content of the raw reads, which were subsequently filtered by the platform to generate clean reads [26]. Transcript assembly was performed using StringTie v3.0.3 (http://ccb.jhu.edu/software/stringtie/) (accessed on 13 October 2025) to obtain unigenes [27]. The unigenes were compared to six major databases (NR, Swiss-Prot, Pfam, EggNOG, GO and KEGG).

The TPM (Transcripts Per Million reads) values of unigene were quantified using RSEM v1.3.2 software [28]. Differentially expressed genes (DEGs) were identified using DESeq2 [29], with genes meeting the criteria of FDR < 0.05 and |log_2_(FC)| ≥ 1 considered as DEGs. Venn analysis was performed to identify common DEGs, which were subsequently subjected to functional annotation and enrichment analysis using the GO and KEGG databases.

#### 2.2.8. Metabolic Analysis

For plant material extraction, 100 mg solid samples were weighed and transferred into 2 mL centrifuge tubes (each with one 6 mm diameter grinding bead), followed by 800 μL extraction solution (methanol:water = 4:1, *v*/*v*) containing four internal standards (including 0.02 mg/mL L-2-chlorophenylalanine). Samples were ground at −10 °C (50 Hz) for 6 min, ultrasonically extracted at 5 °C (40 kHz) for 30 min, incubated at −20 °C for 30 min, centrifuged at 13,000× *g* (4 °C) for 15 min, and the supernatant was transferred to injection vials. For 100 μL liquid samples, 400 μL extraction solution (acetonitrile:methanol = 1:1, *v*/*v*) with the same internal standards was added, vortexed for 30 s, ultrasonically extracted at 5 °C (40 kHz) for 30 min, incubated at −20 °C for 30 min, and centrifuged at 13,000× *g* (4 °C) for 15 min. The supernatant was dried under nitrogen, reconstituted with 100 μL acetonitrile:water (1:1, *v*/*v*), ultrasonically extracted at 5 °C (40 kHz) for 5 min, and centrifuged again at 13,000× *g* (4 °C) for 10 min, and the supernatant was transferred to injection vials. QC samples were prepared by mixing equal volumes of all sample metabolites, with one inserted every 5–10 samples to assess analytical repeatability. LC-MS analysis was performed on a UHPLC-Q Exactive system (Thermo Fisher Scientific, Waltham, MA, USA) using BEH C18, BEH Amide, and HSS T3 columns (Waters Corporation, United States) (100 mm × 2.1 mm i.d., 1.7/1.7/1.8 μm, respectively) at 0.40 mL/min and 40 °C. Mass spectra were collected in positive/negative ion modes (*m*/*z* 50–1200 for C18/Amide, 70–1050 for T3), with ion spray voltages (3500 V/+; −3000 V/−), sheath gas (50 psi for C18/Amide, 50 arb for T3), auxiliary gas (13 psi for C18/Amide, 13 arb for T3), ion source temperature (450 °C), and cyclic collision energy (20–40–60 V).

Samples for metabolome analysis were the same as those for transcriptome sequencing with 6 biological replicates. The sample processing, extraction, and metabolite detection for metabolome analysis were conducted by Shanghai Majorbio Bio-Pharm Biotechnology Co., Ltd. (Shanghai, China) following their standard procedures. Raw data underwent missing value imputation and normalization prior to multivariate analysis using Principal Component Analysis (PCA) and Partial Least Squares-Discriminant Analysis (PLS-DA). Differential accumulation of metabolites (DAMs) was identified based on *p*_value < 0.05 and VIP_pred_OPLS-DA > 1. KEGG pathway mapping was subsequently performed to associate DAMs with biochemical pathways.

#### 2.2.9. Combined Transcriptomic and Metabolomic Analysis

Based on the screening results of the DEGs and DAMs (*p* adjust < 0.05), transcriptomic and metabolomic datasets were integrated through KEGG pathway mapping to explore regulatory mechanisms.

#### 2.2.10. Statistical Analysis

All results in this study were performed in more than three replicates. Data were statistically analyzed and plotted using *t*-test and one-way ANOVA in SPSS Statistics 27 software. In addition, analysis of transcriptomic and metabolomic data was performed on the MajorBio cloud platform (www.MajorBio.com).

## 3. Results

### 3.1. Bioinformatics and Expression Pattern Analysis of MsCKX5 Gene

**Figure 1:**
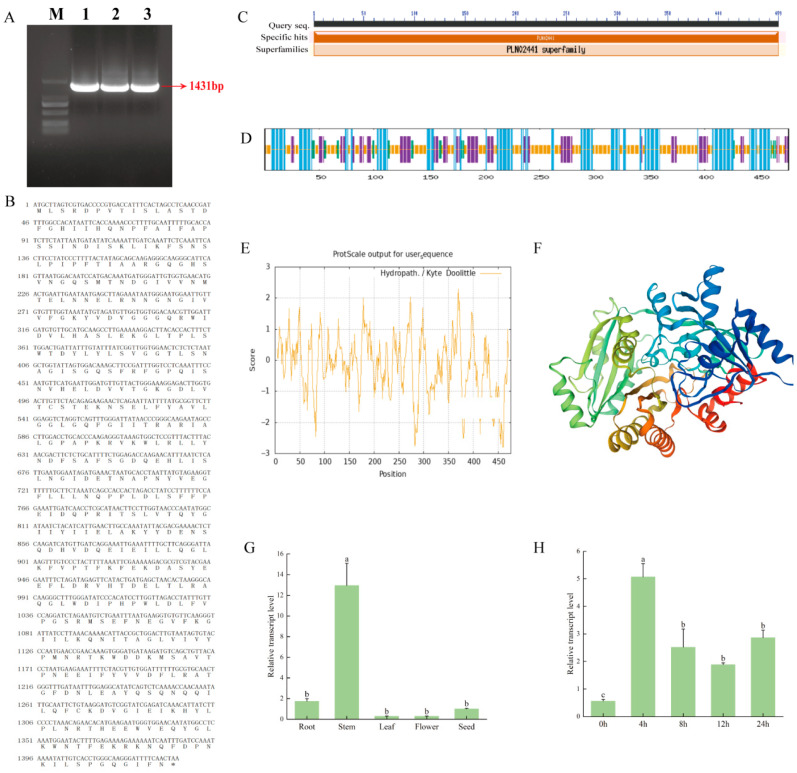
Molecular cloning, bioinformatic characterization, and expression profiling of *MsCKX5* in alfalfa. (**A**) PCR amplification of the full-length coding sequence from ‘Zhongmu No. 1’. (**B**) Nucleotide and deduced amino acid sequences. (**C**) Conserved domain architecture. (**D**) GRAVY-based hydropathy analysis. (**E**) SOPMA-predicted secondary structure. (**F**) SWISS-MODEL-predicted tertiary structure. (**G**) qRT-PCR-based tissue expression profiling. (**H**) Time-course expression under cold stress. Note: The lowercase letters in (**G**,**H**) represent significance.

The *MsCKX5* gene (GenBank: PV934228) was isolated from ‘Zhongmu No. 1’ alfalfa and subjected to bioinformatic characterization. The full-length cDNA spanned 1431 bp (Figure 1A), encoding 477 amino acids (Figure 1B). NCBI protein BLAST identified conserved *CKX5* domains, confirming its membership in the *CKX* family and the presence of the PLN02441 superfamily domain (Figure 1C). The deduced protein exhibited a molecular weight of 53.64 kDa, a theoretical pI of 5.11, and a molecular formula of C_2439_H_3738_N_634_O_715_S_8_. Leucine (10.3%) was the most abundant residue, followed by serine (6.5%), asparagine (6.5%), glutamic acid (5.9%), threonine (5.0%), proline (4.8%), lysine (4.6%), and alanine (4.2%). With a GRAVY value of −0.192, an aliphatic index of 93.76, and an instability index of 38.56, MsCKX5 was predicted as a stable hydrophilic protein (Figure 1D). Secondary structure prediction via SOPMA indicated 31.30% α-helix, 4.62% β-turn, 20.38% extended strand, and 43.70% random coil in MsCKX5 (Figure 1E). Tertiary structure modeling using SWISS-MODEL corroborated these findings (Figure 1F). qRT-PCR analysis revealed ubiquitous *MsCKX5* expression across all tested tissues, with stems showing the highest transcript level (~13-fold relative to seeds) (Figure 1G). Low-temperature stress significantly induced the expression of *MsCKX5* at all time points, peaking at 4 h post-treatment (Figure 1H).

### 3.2. MsCKX5 Overexpression Enhances the Cold Resistance of A. thaliana

**Figure 2:**
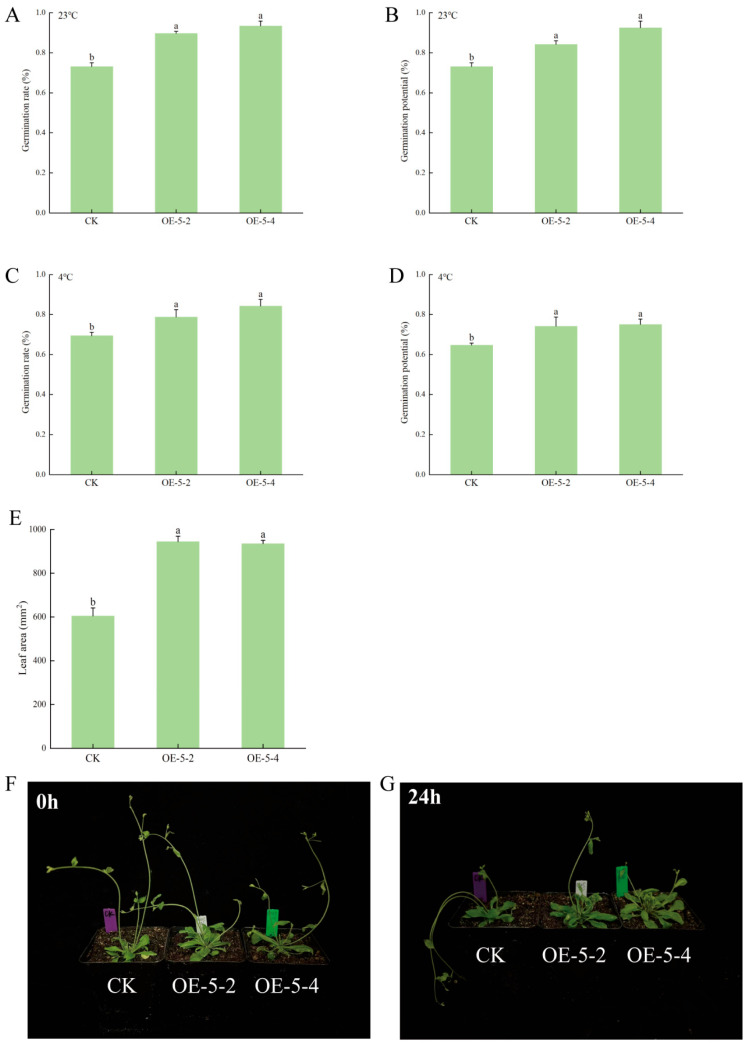
Seed Vigor, leaf area and phenotypic identification of *MsCKX5* transgenic *Arabidopsis*. (**A**–**D**) Germination rate and germination energy of *MsCKX5* transgenic *Arabidopsis*. (**E**) Leaf area of *MsCKX5* transgenic *Arabidopsis*. (**F**,**G**) Phenotypic identification of *MsCKX5* transgenic *Arabidopsis*. Note: The lowercase letters above the graphs represent significance.

To assess the role of *MsCKX5* in cold resistance, Agrobacterium-mediated transformation was employed to generate *MsCKX5*-overexpressing *Arabidopsis* lines (OE-5-2 and OE-5-4). Both wild-type (CK) and transgenic lines were subjected to low-temperature treatment (4 °C) for 0 and 24 h. Under optimal (23 °C) and low-temperature conditions (4 °C), overexpression lines exhibited significantly higher germination rates and vigor than those of CK (*p* < 0.05) (Figure 2A–D). Under normal conditions, transgenic lines showed delayed bolting (Figure 2F) and increased leaf area (*p* < 0.05, Figure 2E) compared to CK. After 24 h cold stress, CK displayed more severe wilting than *MsCKX5*-overexpressing lines (Figure 2G). These results demonstrate that *MsCKX5* overexpression enhances *Arabidopsis* cold tolerance.

**Figure 3:**
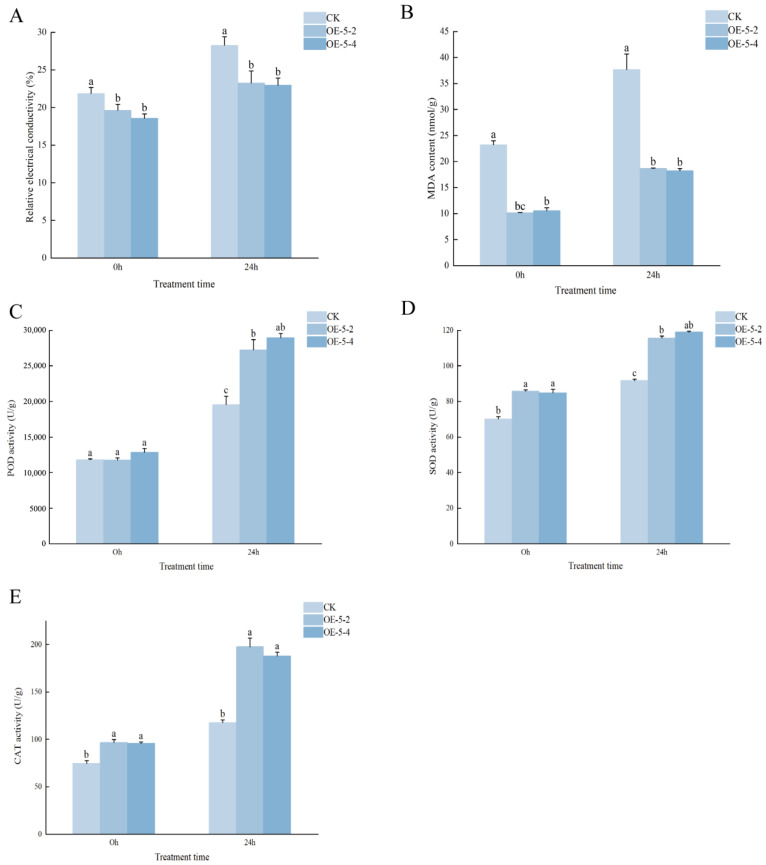
Effects of low-temperature stress on physiological indices of overexpressed lines (OEs) and wild-type (CK) plants. (**A**) Relative electrical conductivity of *Arabidopsis* overexpressing *MsCKX5* before and after low-temperature stress. (**B**) The MDA content of *Arabidopsis* overexpressing *MsCKX5* before and after low-temperature stress. (**C**) The POD activity of *Arabidopsis* overexpressing *MsCKX5* before and after low-temperature stress. (**D**) The SOD activity of *Arabidopsis* overexpressing *MsCKX5* before and after low-temperature stress. (**E**) The CAT activity of *Arabidopsis* overexpressing *MsCKX5* before and after low-temperature stress. Note: The lowercase letters above the graphs represent significance.

To further elucidate the mechanism underlying *MsCKX5*-mediated cold resistance, relative electrolyte leakage and malondialdehyde (MDA) content were quantified. Prior to cold stress, relative electrolyte leakage was similar between wild-type and *MsCKX5*-overexpressing lines (Figure 3A). Following 24 h treatment at 4 °C, a 6.39% increase in leakage was observed in CK, whereas increases of only 3.63% and 4.39% were detected in OE-5-2 and OE-5-4 lines, respectively, indicating reduced membrane damage in transgenic lines. Under control conditions, significantly lower MDA levels were measured in transgenic lines compared to CK (*p* < 0.05, Figure 3B). After 24 h of cold stress, MDA content was elevated in both genotypes; however, this increase was markedly attenuated in *MsCKX5*-overexpressing lines. These results suggest that membrane lipid peroxidation is mitigated by *MsCKX5*, thereby enhancing cellular integrity under cold stress.

Simultaneously, antioxidant enzyme activities were quantified in plants subjected to cold stress. Prior to stress, no significant difference in peroxidase (POD) activity was observed between CK and *MsCKX5*-overexpressing lines (Figure 3C). Following cold stress, POD activity was elevated in both genotypes, with CK levels significantly lower than those of transgenic lines (*p* < 0.05). Superoxide dismutase (SOD) activity was also significantly lower in CK than that of transgenic lines before stress (Figure 3D) and remained so after stress despite increases in both genotypes (*p* < 0.05). Catalase (CAT) activity followed a similar pattern: lower in CK before stress, with a markedly attenuated increase after stress compared to transgenic lines (Figure 3E). These results indicate that *MsCKX5* enhances antioxidant enzyme activity, thereby maintaining redox homeostasis, alleviating oxidative damage, and improving cold tolerance in *Arabidopsis*.

### 3.3. Transcriptome Analysis

#### 3.3.1. Transcriptome Sequencing Quality Assessment

Transcriptome sequencing was performed on wild-type (CK) and *MsCKX5*-overexpressing (OE-5-2 and OE-5-4) *Arabidopsis* plants subjected to cold stress for 0 and 24 h. Raw reads per sample ranged from 37.89 to 52.05 million, with error rates of 0.01%, Q30 values > 96%, and GC contents of 42.2–45.32% (Table 2).

#### 3.3.2. Analysis of Differential Gene Expression

**Figure 4:**
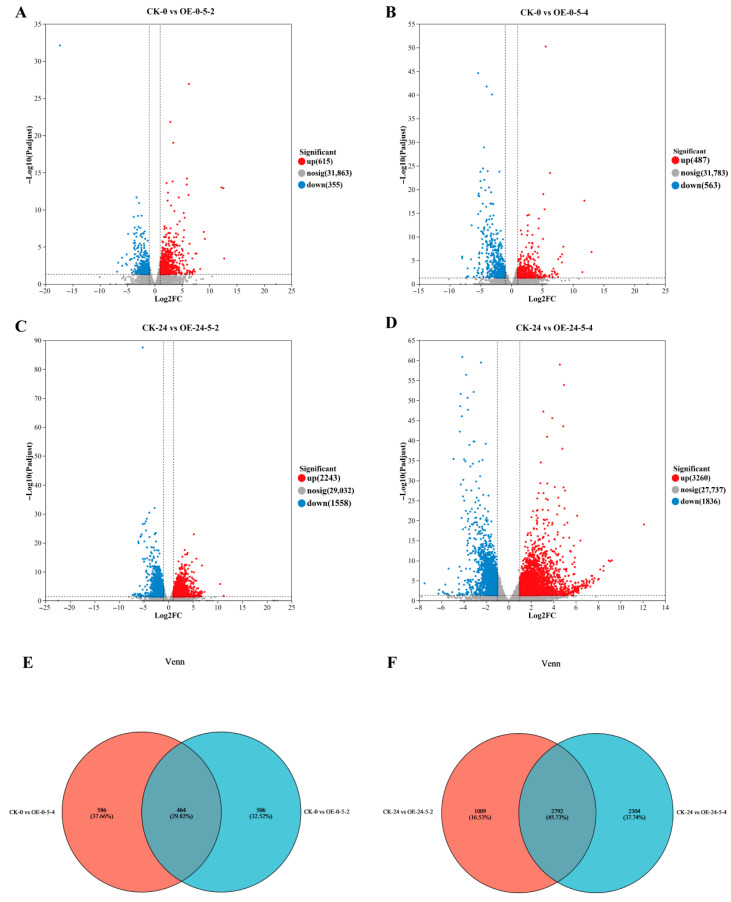
Transcriptome analysis of *MsCKX5*-overexpressing and CK plants. (**A**) Number of DEGs in CK-0 vs. OE-0-5-2 of *MsCKX5* transgenic *Arabidopsis thaliana*. (**B**) Number of DEGs in CK-0 vs. OE-0-5-4 of *MsCKX5* transgenic *A. thaliana*. (**C**) Number of DEGs in CK-24 vs. OE-24-5-2 of *MsCKX5* transgenic *A. thaliana*. (**D**) Number of DEGs in CK-24 vs. OE-24-5-4 of *MsCKX5* transgenic *A. thaliana*. (**E**) Venn map of DEGs in CK-0 vs. OE-0-5-2 and CK-0 vs. OE-0-5-4 of *MsCKX5* transgenic *A. thaliana*. (**F**) Venn map of DEGs in CK-24 vs. OE-24-5-2 and CK-24 vs. OE-24-5-4 of *MsCKX5* transgenic *A. thaliana*.

Using |log_2_(FC)| ≥ 2 and FDR ≤ 0.05 as thresholds, DEGs were identified between two *MsCKX5*-overexpressing lines (OE-5-2 and OE-5-4) and wild-type (CK) at 0 and 24 h of cold stress. At 0 h, 1050 DEGs (487 upregulated, 563 downregulated) were detected in OE-0-5-2 vs. CK-0, and 970 DEGs (615 upregulated, 355 downregulated) in OE-0-5-4 vs. CK-0. Venn analysis revealed 1556 total DEGs, with 464 shared and 506/586 unique to OE-0-5-2/OE-0-5-4, respectively (Figure 4E). At 24 h, 3801 DEGs (2243 upregulated, 1558 downregulated) were identified in OE-24-5-2 vs. CK-24, and 5096 DEGs (3260 upregulated, 1836 downregulated) in OE-24-5-4 vs. CK-24. Venn diagram analysis of OE-24-5-2 vs. CK-24 and OE-24-5-4 vs. CK-24 revealed 6150 DEGs, with 2792 shared and 1009/2304 unique to OE-24-5-2/OE-24-5-4, respectively (Figure 4F).

#### 3.3.3. GO and KEGG Enrichment Analysis of Differentially Expressed Genes

**Figure 5:**
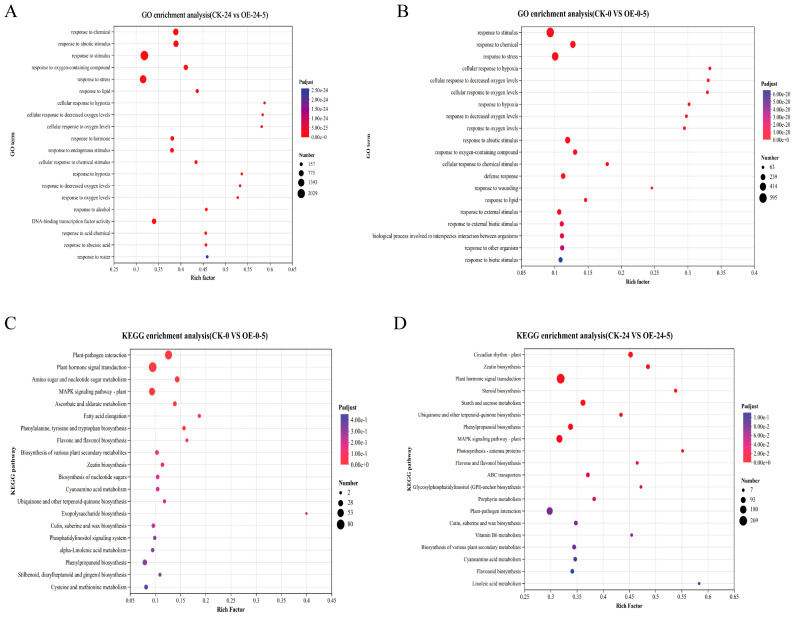
Transcriptome analysis of *MsCKX5*-overexpressing and CK plants. (**A**) GO enrichment analysis of DEGs of CK-0 vs. OE-0-5 in *MsCKX5* transgenic *A. thaliana*. (**B**) GO enrichment analysis of DEGs of CK-24 vs. OE-24-5 in *MsCKX5* transgenic *A. thaliana*. (**C**) KEGG enrichment analysis of DEGs of CK-0 vs. OE-0-5 in *MsCKX5* transgenic *A. thaliana*. (**D**) KEGG enrichment analysis of DEGs of CK-24 vs. OE-24-5 in *MsCKX5* transgenic *A. thaliana*.

To elucidate the functional significance of transcriptome alterations, GO and KEGG pathway enrichment analyses were conducted on the identified DEGs. Transcriptome data from two *MsCKX5*-overexpressing lines (OE-5-2 and OE-5-4) and wild-type (CK) were subjected to GO enrichment analysis at 0 and 24 h of cold stress. The analysis revealed 510 enriched GO terms in CK-0 vs. OE-0-5, comprising 387 biological process (BP), 20 Cellular Component (CC), and 103 Molecular Function (MF) terms. The top 20 BP terms included response to stimulus, response to chemical, response to stress, cellular response to hypoxia, cellular response to decreased oxygen levels, cellular response to oxygen levels, response to hypoxia, response to decreased oxygen levels, response to oxygen levels, response to abiotic stimulus, response to oxygen-containing compound, cellular response to chemical stimulus, defense response, response to wounding, response to lipid, response to external stimulus, response to external biotic stimulus, biological processes involved in interspecies interactions, response to other organisms, and response to biotic stimulus (Figure 5A). In CK-24 vs. OE-24-5, 951 GO terms were enriched, comprising 641 Biological Process (BP), 67 Cellular Component (CC), and 243 Molecular Function (MF) terms. The top 20 significantly enriched terms are shown in Figure 5B. Of these, 19 were BP terms, including responses to stress, lipids, hypoxia, decreased oxygen levels, hormones, endogenous stimuli, chemical stimuli, alcohol, acidic chemicals, abscisic acid, and water. The sole MF term was DNA-binding transcription factor activity.

KEGG pathway enrichment analysis was performed with *p* < 0.05 as the significance threshold. In CK-0 vs. OE-0-5, 107 pathways were enriched, with 7 significantly enriched among the top 20 displayed (Figure 5C): plant–pathogen interaction, plant hormone signal transduction, MAPK signaling pathway—plant, amino sugar and nucleotide sugar metabolism, ascorbate and aldarate metabolism, phenylalanine, tyrosine and tryptophan biosynthesis, and fatty acid elongation. In CK-24 vs. OE-24-5, 135 pathways were enriched, with 13 significantly enriched among the top 20 (Figure 5D): plant hormone signal transduction, MAPK signaling pathway—plant, phenylpropanoid biosynthesis, circadian rhythm—plant, zeatin biosynthesis, ubiquinone and other terpenoid–quinone biosynthesis, starch and sucrose metabolism, photosynthesis-antenna proteins, flavone and flavonol biosynthesis, ABC transporters, glycosylphosphatidylinositol (GPI)-anchor biosynthesis, porphyrin metabolism, and steroid biosynthesis.

### 3.4. Metabolome Analysis

#### 3.4.1. Sample Comparison Analysis

**Figure 6:**
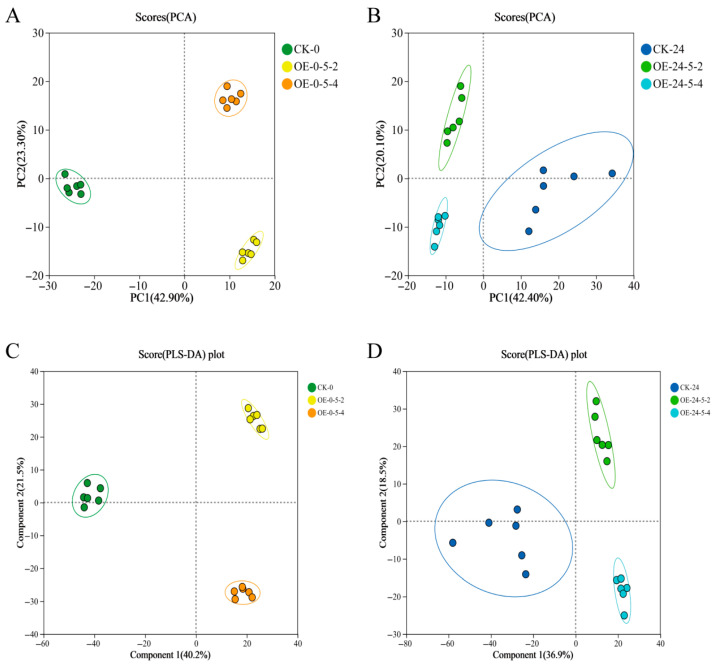
Sample analysis of metabolome. (**A**) Sample PCA analysis of CK-0 vs. OE-0-5. (**B**) Sample PCA analysis of CK-24 vs. OE-24-5. (**C**) Sample PLS-DA analysis of CK-0 vs. OE-0-5. (**D**) Sample PLS-DA analysis of CK-24 vs. OE-24-5.

Principal Component Analysis (PCA) was performed on wild-type (CK) and two *MsCKX5*-overexpressing lines (OE-5-2 and OE-5-4). At 0 h of cold stress, 42.90% and 23.30% of the variance were explained by PC1 and PC2, respectively; at 24 h, these values were 42.40% and 20.10% (Figure 6A,B). Tight clustering of the six biological replicates within each group was observed, indicating high reproducibility. For Partial Least Squares-Discriminant Analysis (PLS-DA), Q2 (cum) values of 0.974 and 0.976, and R2Y values of 0.992 and 0.994, were obtained for 0 h and 24 h, respectively (Figure 6C,D), demonstrating robust model explanatory power and predictive capability.

#### 3.4.2. Analysis of Differential Metabolite Expression

**Figure 7:**
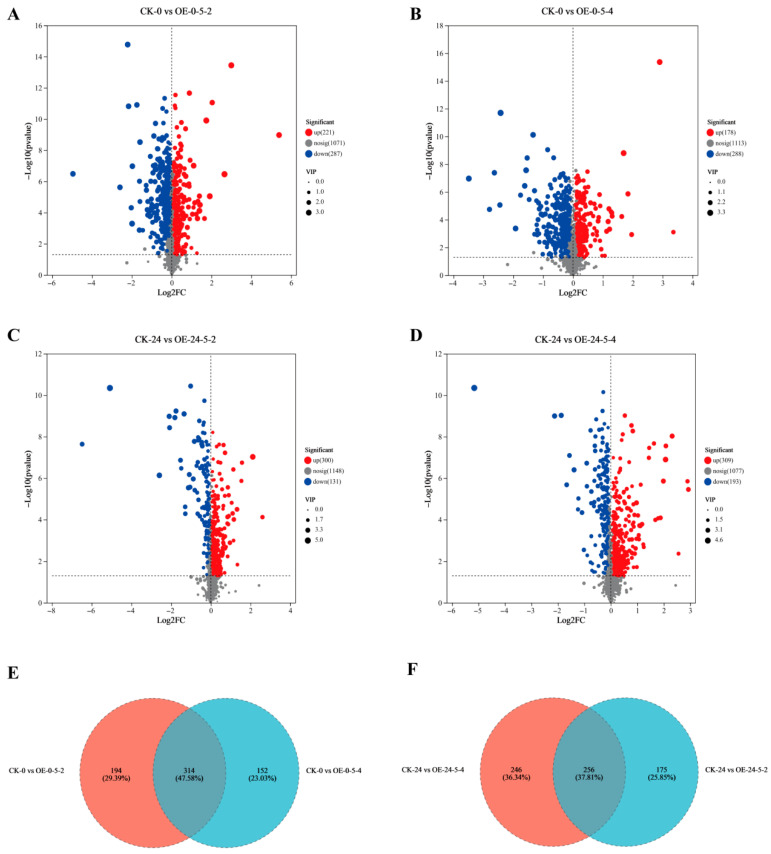
Transcriptome analysis of *MsCKX5*-overexpressing and CK plants. (**A**) Number of DAMs in CK-0 vs. OE-0-5-2 of *MsCKX5* transgenic *A. thaliana*. (**B**) Number of DAMs in CK-0 vs. OE-0-5-4 of *MsCKX5* transgenic *A. thaliana*. (**C**) Number of DAMs in CK-24 vs. OE-24-5-2 of *MsCKX5* transgenic *A. thaliana*. (**D**) Number of DAMs in CK-24 vs. OE-24-5-4 of *MsCKX5* transgenic *A. thaliana*. (**E**) Venn map of DAMs in CK-0 vs. OE-0-5-2 and CK-0 vs. OE-0-5-4 of *MsCKX5* transgenic *A. thaliana*. (**F**) Venn map of DAMs in CK-24 vs. OE-24-5-2 and CK-24 vs. OE-24-5-4 of *MsCKX5* transgenic *A. thaliana*.

Differential metabolites were identified between two *MsCKX5*-overexpressing lines (OE-5-2 and OE-5-4) and wild-type (CK) using thresholds of |log_2_(FC)| ≥ 2 and FDR ≤ 0.05 at 0 h and 24 h of cold stress. At 0 h, 508 differential metabolites (221 upregulated, 287 downregulated) were detected in OE-0-5-2 vs. CK-0, and 466 (178 upregulated, 288 downregulated) in OE-0-5-4 vs. CK-0 (Figure 7A,B). Venn analysis revealed 660 differential metabolites in total, with 314 shared and 194/152 unique to OE-0-5-2/OE-0-5-4, respectively (Figure 7E). At 24 h of cold stress, 431 differential metabolites (300 upregulated, 131 downregulated) were detected in OE-24-5-2 vs. CK-24, and 502 (309 upregulated, 193 downregulated) in OE-24-5-4 vs. CK-24 (Figure 7C,D). Venn analysis revealed 677 differential metabolites in total, with 256 shared and 246/175 unique to OE-24-5-2/OE-24-5-4, respectively (Figure 7F).

#### 3.4.3. KEGG Enrichment Analysis of Differential Metabolites

**Figure 8:**
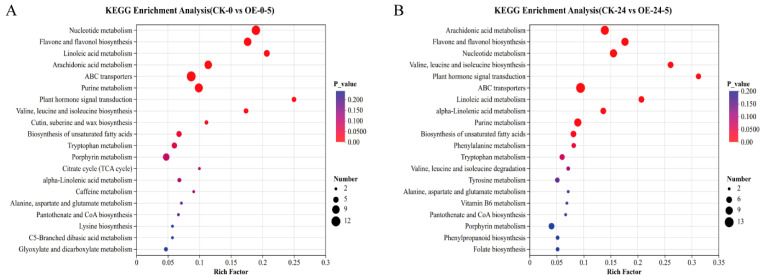
Metabolome analysis of *MsCKX5*-overexpressing lines and CK plants. (**A**) KEGG enrichment analysis of DAMs of CK-0 vs. OE-0-5 in *MsCKX5* transgenic *A. thaliana*. (**B**) KEGG enrichment analysis of DAMs of CK-24 vs. OE-24-5 in *MsCKX5* transgenic *A. thaliana*.

Metabolomic analysis was performed on wild-type (CK) and two *MsCKX5*-overexpressing lines (OE-5-2 and OE-5-4). KEGG pathway enrichment analysis of differential metabolites was conducted with *p* < 0.05 as the significance threshold. In CK-0 vs. OE-0-5, 73 pathways were enriched, with 10 significantly enriched among the top 20 displayed (Figure 8A): biosynthesis of unsaturated fatty acids, cutin, suberine and wax biosynthesis, valine, leucine and isoleucine biosynthesis, plant hormone signal transduction, purine metabolism, ABC transporters, arachidonic acid metabolism, linoleic acid metabolism, flavone and flavonol biosynthesis, and nucleotide metabolism. In CK-24 vs. OE-24-5, 76 pathways were enriched, with 11 significantly enriched among the top 20 (Figure 8B): phenylalanine metabolism, biosynthesis of unsaturated fatty acids, purine metabolism, alpha-linolenic acid metabolism, linoleic acid metabolism, ABC transporters, plant hormone signal transduction, valine, leucine and isoleucine biosynthesis, nucleotide metabolism, flavone and flavonol biosynthesis, and arachidonic acid metabolism.

### 3.5. Combined Analysis of Transcriptome and Metabolome

#### 3.5.1. KEGG Enrichment Analysis

**Figure 9:**
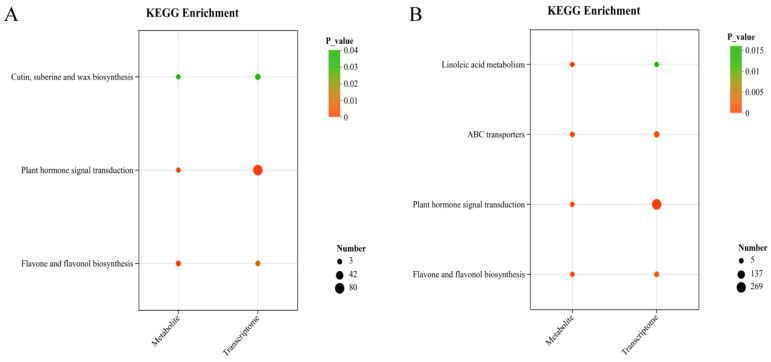
Integrated transcriptomic and metabolomic analysis of *MsCKX5*-overexpressing lines and CK plants. (**A**) Integrated KEGG enrichment analysis of DEGs and DAMs of CK-0 vs. OE-0-5 in *MsCKX5* transgenic *A. thaliana*. (**B**) Integrated KEGG enrichment analysis of DAMs of CK-24 vs. OE-24-5 in MsCKX5 transgenic *A. thaliana*.

To outline the metabolic network regulated by *MsCKX5*, we combined transcriptomic and metabolomic data using KEGG pathway mapping. Joint analysis revealed that the DEGs and DAMs were concurrently enriched in plant hormone signal transduction, as well as flavone and flavonol biosynthesis (Figure 9).

#### 3.5.2. Key Pathway Analysis

**Figure 10:**
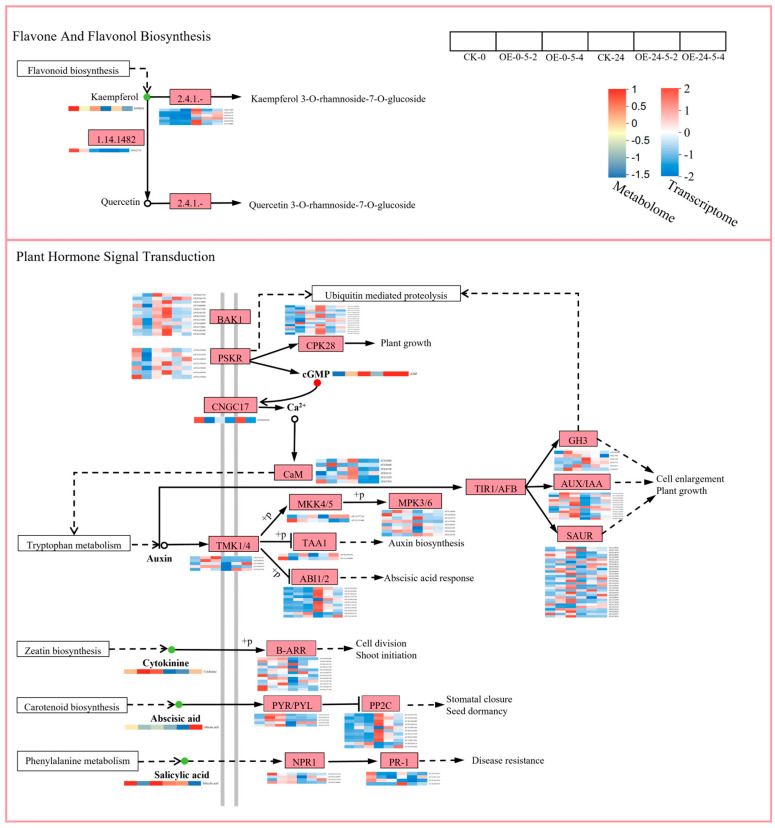
Combined metabolomic and transcriptomic analysis of key pathway.

To investigate the response of *Arabidopsis* overexpressing *MsCKX5* to cold stress, the plant hormone signal transduction and flavone and flavonol biosynthesis pathways were examined in wild-type (CK) and two *MsCKX5*-overexpressing lines (OE-5-2 and OE-5-4) at 0 h and 24 h. In the hormone pathway, 156 DEGs were enriched (Figure 10). The cytokinin signaling response factors (B-ARRs), salicylic acid signaling response factors (NPR1 and PR-1), and abscisic acid signaling response factors (PYR/PYL) were predominantly downregulated following low-temperature stress. Notably, the wild-type exhibited a more pronounced downregulatory trend compared with the overexpression lines, suggesting that the overexpression lines possess enhanced stress responsiveness. The abscisic acid signaling response factors (PP2Cs) exhibited differential expression patterns under low-temperature stress, with 12 genes upregulated and 1 gene downregulated, indicating activation of the ABA signaling pathway. Conversely, the majority of auxin signaling response factors (TMK1/4, AUX/IAA, GH3, and SAUR) were downregulated following low-temperature stress. This downregulation suggests that auxin receptors and response repressors are suppressed under cold conditions, which may contribute to the attenuation of auxin-mediated growth processes, thereby enhancing plant stress tolerance. Transmembrane receptors (BAK1, CNGC17, PSKR), calcium signal regulatory modules (CaM, CPK28), and downstream MAPK cascades (MKK4/5, MPK3/6) were differentially expressed, mediating extracellular-to-intracellular signal transduction. Cytokinin, abscisic acid, and salicylic acid levels were decreased, whereas cGMP was increased. In the flavone and flavonol pathway, seven DEGs were co-enriched, comprising one downregulated key enzyme of the phenylpropanoid pathway (1.14.18.2) and six upregulated flavonol glycosyltransferases (2.4.1.-). Kaempferol, a core secondary metabolite of the flavonol class, exhibited a downregulatory trend in the flavonoid and flavonol biosynthetic pathway, which facilitated the synthesis of kaempferol-3-O-rhamnoside-7-O-glucoside (2.4.1.-) and consequently enhanced the cold tolerance of transgenic plants.

### 3.6. RT-PCR Validation

**Figure 11:**
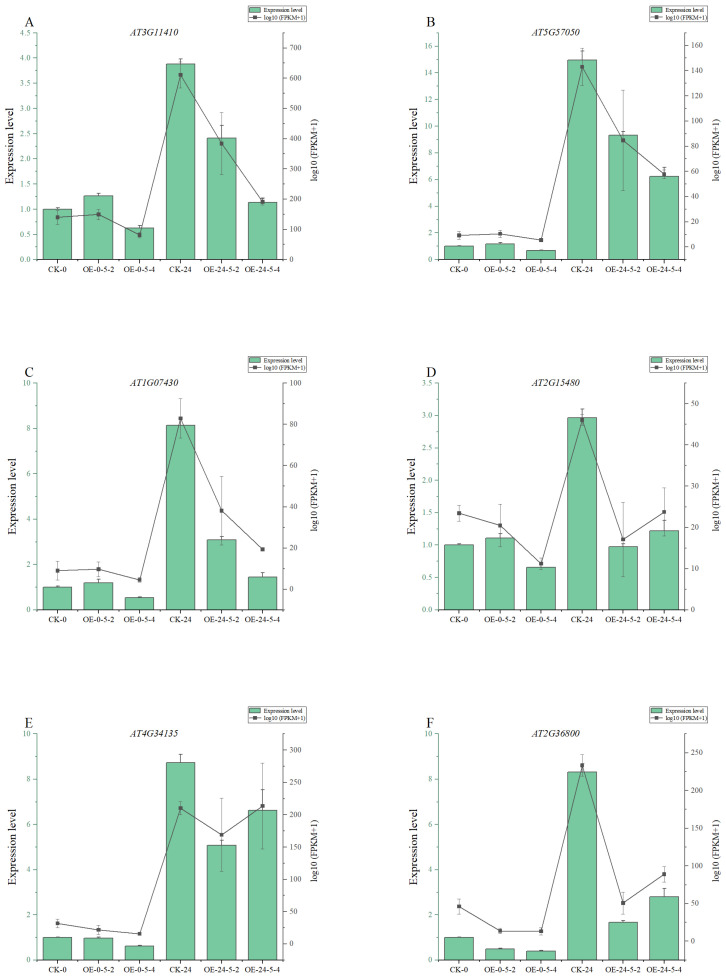
RT-PCR validation of differential expressed genes. The vertical axis represents gene expression levels and log10 (FPKM) values. The horizontal axis represents different treatments of alfalfa. Bar graphs represent RT-PCR, while line graphs represent RNA-seq. (**A**–**F**) Show the analysis of the relative expression level and transcriptome expression level of *AT3G11410*, *AT5G57050*, *AT1G07430*, *AT2G15480*, *AT4G34135*, *AT2G36800*.

To verify the reproducibility and accuracy of differential expression results from RNA-Seq, we randomly selected seven genes (*AT4G26080*, *AT3G11410*, *AT5G57050*, *AT1G07430*, *AT2G15480*, *AT2G36750*, *AT4G34135*, *AT2G36800*) from KEGG significantly enriched pathways for RT-PCR validation. Although the fold changes measured by qRT-PCR for these genes did not exactly match the RNA-Seq fold changes, the direction of expression change was consistent between methods. This concordance indicates that the transcriptome sequencing data are reliable (Figure 11).

## 4. Discussion

The *CKX* gene family has been extensively characterized in *A. thaliana* [16], soybean [19] and rice [18]. Studies have shown that this family regulates plant growth, development, and responses to environmental stresses. Based on previous identification of *CKX* genes in *M. sativa*, we isolated *MsCKX5*, which responds to cold stress. Cloning and sequence analysis revealed a full-length cDNA of 1431 bp, encoding a 477-amino-acid protein containing the PLN02441 superfamily domain; *MsCKX5* was characterized as a stable hydrophilic protein. In cucumber [30], *CsCKX* genes were prominently expressed in roots and male flowers. Under salt stress, *CsCKX* expression declined over time in leaves (except *CsCKX4* and *CsCKX8*), while all members were upregulated in stems. *MsCKX5* showed highest expression in stems, followed by roots. Under cold stress, *MsCKX5* was significantly upregulated at all time points, peaking at 4 h.

Cold stress impacts membrane lipid peroxidation and cell membrane permeability in plants; changes in MDA content and relative electrolyte leakage are direct indicators of oxidative damage and membrane permeability alterations [31,32]. Plants activate antioxidant enzyme systems to combat oxidative stress under cold stress, resulting in altered antioxidant enzyme activity that mitigates membrane lipid peroxidation and elevated membrane permeability, thus maintaining membrane integrity [33]. The extent of damage is closely associated with plant phenotype [34]. To investigate the role of *MsCKX5* in cold resistance, phenotypes and physiological parameters of wild-type (CK) and transgenic *Arabidopsis* (OE-5-2, OE-5-4) were evaluated before and after cold treatment. Under normal and cold conditions, germination rates of *MsCKX5*-overexpressing lines (OE-5-2 and OE-5-4) were significantly higher than those of wild-type (CK). After 45 days, the leaf area of transgenic lines was significantly larger than that of wild-type. Following cold stress, wild-type exhibited more severe wilting than transgenic lines. CK also exhibited significantly greater increases in MDA content and relative electrolyte leakage than transgenic lines (*p* < 0.05). SOD, POD, and CAT activities were significantly altered in response to cold acclimation. These results suggest that *MsCKX5* overexpression mitigates cold stress-induced injury.

Integrated transcriptomic and metabolomic analysis demonstrated that transgenic *A. thaliana* responded to cold stress through modulation of flavone and flavonol biosynthesis and plant hormone signal transduction. Upregulation of key enzyme genes in these pathways, together with accumulation of glycosylated flavonol metabolites, mitigated oxidative and osmotic damage through scavenging of ROS, membrane stabilization, and enhanced osmotic adjustment [35,36]. The phytohormone signal transduction pathway regulated the balance between plant growth and defense through modification of auxin signaling, activation of the abscisic acid (ABA) pathway, integration of cytokinin and salicylic acid signaling cascades, and enhancement of cold resistance mechanisms via the mitogen-activated protein kinase (MAPK) cascade and calcium signal transduction. Low temperatures induced upregulation of auxin biosynthetic genes (TAA1, TMK1/4) in transgenic *Arabidopsis*, which promoted auxin synthesis via the tryptophan metabolic pathway. Simultaneously, pivotal signaling components (TIR1/AFB, AUX/IAA, SAUR) were activated, maintaining root growth and morphogenesis through regulation of cell elongation and division, as reported by Steffen et al. [37]. Zhao et al. demonstrated that continuous activation of the MKK4/5-MPK3/6 cascade decreased CBF gene expression and increased susceptibility to freezing injury, suggesting a negative regulatory role of this cascade in cold response [38]. This study revealed that auxin interacted with the MAPK pathway through MKK4/5 and MPK3/6, indicating potential collaboration between auxin and the MAPK cascade to enhance expression of CBF/DREB family cold resistance genes, thereby mitigating cold stress effects.

The ABA biosynthetic pathway, a key phytohormone pathway for cold stress response, showed upregulation of key genes in transcriptomic data. These genes interacted with ABA receptors (PYR/PYL) and downstream PP2C genes. Inhibition of PP2C-mediated negative regulation of SnRK2 stimulated expression of ABRE-driven cold resistance genes (RD29A, COR15A), enhancing cell membrane stability and osmotic adjustment capacity [39]. Jeon et al. demonstrated that low temperatures induced A-ARR expression through core components of the two-component system (TCS) in a cytokinin-independent manner, which regulated freezing resistance [40]. In this study, A-ARR was upregulated in the cytokinin biosynthetic pathway (zeatin biosynthesis), suggesting its role in maintaining plant vigor under cold stress through regulation of cell division and bud formation [41]. Mohd Saleem (2020) reported that salicylic acid synthesis activated multiple signaling pathways, mitigating membrane damage, metabolic disorders, and oxidative stress induced by low temperatures through coordinated regulation of antioxidant systems, osmotic adjustment, and expression of molecular chaperones and cold resistance-related proteins [42]. Salicylic acid also facilitated crosstalk with ABA, Ca^2+^, and MAPK pathways, thus establishing dynamic equilibrium between stress resistance and growth. The salicylic acid biosynthetic pathway, part of phenylalanine metabolism, was associated with enhanced activities of antioxidant enzymes (SOD, POD, CAT), thereby reducing ROS levels and alleviating membrane lipid peroxidation. This pathway also promoted production of pathogenesis-related (PR) proteins, leading to decreased relative electrolyte leakage and reduced MDA accumulation [43]. Interaction of this pathway with ABA, jasmonic acid (JA), Ca^2+^, and MAPK signaling contributed to coordinated regulation of cold resistance and enhancement of plant defense mechanisms under cold stress. Overall, these results suggest that transgenic *A. thaliana* enhanced cold tolerance primarily through transcriptional regulation of key genes and accumulation of metabolites in plant hormone signal transduction and flavone and flavonol biosynthesis pathways.

## 5. Conclusions

The cold stress-responsive gene *MsCKX5* was cloned from ‘Zhongmu No. 1’ alfalfa. *MsCKX5* overexpression in *A. thaliana* significantly increased leaf area and enhanced cold tolerance. Mechanistically, *MsCKX5* upregulated key flavonol biosynthesis enzymes, promoting kaempferol-3-O-rhamnoside-7-O-glucoside accumulation to maintain intracellular redox homeostasis. It also enhanced SOD, POD, and CAT activities, regulating relative electrolyte leakage and MDA accumulation to alleviate membrane lipid peroxidation and oxidative damage under low temperature. Additionally, *MsCKX5* reinforced cold tolerance by modulating the ABA signaling pathway, where upregulated PP2C negative regulators maintained ABA signaling at an optimal level.

## Figures and Tables

**Table 1:** Primer sequence.

Primers	Primer Sequence (5′-3′)	PCR Product Length
*MsCKX5-F*	ATGCTTAGTCGTGACCC	1431 bp
*MsCKX5-R*	TTAGTTGAAAATCCCTTG	
PN560-*MsCKX5-F*	CAAGCTTCGCCACCATGGAATTCATGCTTAGTCGTGACCCCGTGAC	1431 bp
PN560-*MsCKX5-R*	GTCTTCTGCTTGTCTAGGATCCCGTTGAAAATCCCTTGCCCAG	
*MsCKX5*-F	GACGCACAATCCCACTATCC	1474 bp
pCAMBIA1300-2×35S-EGFP-R	CGCTGAACTTGTGGCCGT	
RT-PCR-*MsCKX5*-F	AAGAGGGCAAGGGCATTCAG	
RT-PCR-*MsCKX5*-R	CAACGTTGTCCACCACCAAC	
RT-PCR-Ms-*β-actin*-F	TTTGAGACTTTCAATGTGCCCGCC	
RT-PCR-Ms-*β-actin*-R	TAGCATGTGGGAGTGCATAACCCT	

**Table 2:** Quality assessment of transcriptome sequencing.

Sample	Raw Reads	Clean Reads	Clean Bases	Error Rate (%)	Q20 (%)	Q30 (%)	GC Content (%)
CK-0-1	44,579,120	44,302,590	6,635,662,128	0.0115	99.39	96.87	42.2
CK-0-2	47,611,802	47,349,726	7,084,886,414	0.0114	99.43	96.95	43.89
CK-0-3	37,899,274	37,701,804	5,633,522,713	0.0115	99.43	96.83	45.05
CK-24-1	46,126,102	45,840,652	6,797,244,316	0.0115	99.41	96.81	44.45
CK-24-2	48,594,390	48,326,566	7,224,806,362	0.0115	99.42	96.83	45
CK-24-3	44,165,926	43,911,146	6,582,273,140	0.0114	99.43	96.89	45.3
OE-0-5-2-1	39,544,066	39,326,256	5,902,176,312	0.0114	99.43	96.88	45.09
OE-0-5-2-2	46,102,720	45,881,146	6,891,411,074	0.0114	99.44	96.95	44.75
OE-0-5-2-3	41,262,736	41,032,984	6,150,922,045	0.0114	99.44	96.96	45.32
OE-0-5-4-1	52,051,552	51,706,008	7,741,937,904	0.0115	99.39	96.72	44.7
OE-0-5-4-2	38,319,918	38,080,670	5,709,310,092	0.0115	99.42	96.84	45.24
OE-0-5-4-3	42,384,152	42,135,810	6,327,259,360	0.0115	99.4	96.71	44.62
OE-24-5-2-1	47,652,284	47,411,784	7,108,291,351	0.0114	99.44	96.89	44.66
OE-24-5-2-2	45,814,698	45,531,028	6,836,797,603	0.0114	99.42	96.89	44.45
OE-24-5-2-3	47,446,448	47,133,708	7,032,630,071	0.0114	99.41	96.91	43.18
OE-24-5-4-1	45,441,758	45,143,146	6,744,629,451	0.0115	99.41	96.85	44.54
OE-24-5-4-2	47,267,328	46,980,344	7,028,629,998	0.0114	99.44	96.94	44.24
OE-24-5-4-3	42,704,490	42,485,612	6,345,025,507	0.0115	99.44	96.86	44.95

## Data Availability

The datasets generated during and analyzed during the current study are available from the corresponding author on reasonable request.
